# Efficacy and Safety of Erenumab in the Preventive and Therapeutic Treatment of Patients Suffering From Migraine

**DOI:** 10.7759/cureus.115403

**Published:** 2026-08-29

**Authors:** Yatri Patel, Siddharth Panchal, Aarav Patel, Vishwa Parker, Harshini S Rathod, Pankhuri Saxena

**Affiliations:** 1 Geriatric Medicine, All India Institute of Medical Sciences Rishikesh, Rishikesh, IND; 2 Gastroenterology, All India Institute of Medical Sciences Rishikesh, Rishikesh, IND; 3 General Practice, All India Institute of Medical Sciences Jodhpur, Jodhpur, IND; 4 Medicine, Smt. NHL (Nathiba Hargovandas Lakhmichand) Municipal Medical College, Ahmedabad, IND

**Keywords:** efficacy, erenumab, meta-analysis, migraine, safety

## Abstract

Migraine is a prevalent and disabling neurological disorder that significantly impairs quality of life and represents a major public health concern worldwide. Erenumab, a fully human monoclonal antibody targeting the calcitonin gene-related peptide (CGRP) receptor, has emerged as a mechanism-based preventive therapy for migraine. This systematic review and meta-analysis evaluated the efficacy and safety of erenumab in randomized controlled trials (RCTs). Six independent RCTs involving 3,403 randomized participants were included in the quantitative synthesis. Outcome-specific denominators varied across analyses because individual trials used different efficacy and safety analysis populations and reported different outcomes. The primary outcome was the proportion of patients achieving a ≥50% reduction in monthly migraine days after 12 weeks of treatment, while secondary outcomes included changes in monthly migraine days and the incidence of adverse events. Pooled analysis demonstrated that erenumab significantly increased the likelihood of achieving a ≥50% responder rate compared with placebo (RR = 1.660, 95% CI: 1.364-2.020; p < 0.001). Erenumab also produced a significantly greater reduction in monthly migraine days compared with placebo (MD = −1.51 days/month, 95% CI: −2.03 to −0.99; p < 0.001), although substantial between-study heterogeneity was observed (I² = 76.4%). Evidence for lower-dose regimens was limited and less consistent across studies, whereas the 70-mg and 140-mg regimens demonstrated significant benefit in the included trials. The overall incidence of adverse events was comparable between the erenumab and placebo groups (RR = 0.968, 95% CI: 0.894-1.048), with low-to-moderate heterogeneity (I² = 33.2%). Similarly, no statistically significant difference was observed in serious adverse events (RR = 0.791, 95% CI: 0.463-1.349; p = 0.389; I² = 0%). These findings suggest that erenumab is effective for migraine prevention and was not associated with a statistically significant increase in adverse or serious adverse events compared with placebo in the included trials. Overall, the available evidence supports erenumab as an effective preventive treatment for patients with episodic or chronic migraine.

## Introduction and background

Migraine is a common neurological disorder and one of the leading causes of disability worldwide, imposing a considerable burden on affected individuals and healthcare systems. It is estimated that nearly one-third of adults aged 18-65 years experience headache disorders, with migraine accounting for approximately one-third of these cases [1]. Based on headache frequency, migraine is classified into episodic migraine (EM) and chronic migraine (CM). EM is characterized by fewer than 15 headache days per month, whereas CM is diagnosed when headache occurs on at least 15 days per month for more than three months, with migraine features on at least eight days per month. Chronic migraine often evolves from episodic migraine and is associated with greater functional impairment and reduced quality of life [2].

Migraine attacks are typically characterized by recurrent episodes of moderate-to-severe headache, frequently accompanied by gastrointestinal symptoms such as nausea and vomiting and sensory disturbances including photophobia, phonophobia, and increased sensitivity to odors. Some patients may also experience visual symptoms, paresthesia, or transient language disturbances. Collectively, these manifestations can interfere substantially with routine activities and adversely affect physical, social, and occupational well-being [3].

Several pharmacological agents, including valproate, propranolol, amitriptyline, and verapamil, are used for migraine prevention [4,5]. Although these treatments have established clinical utility, their use may be limited in some patients by adverse effects, contraindications, tolerability, adherence, and individual variation in treatment response. These limitations have encouraged the development of preventive therapies that specifically target biological mechanisms implicated in migraine pathogenesis.

Growing evidence identifies calcitonin gene-related peptide (CGRP), a 37-amino-acid neuropeptide, as an important mediator of migraine pathophysiology through its role in the trigeminovascular system [6,7]. This understanding has facilitated the development of monoclonal antibodies that selectively inhibit CGRP signaling and provide a mechanism-based approach to migraine prevention [8]. Among these agents, erenumab (AMG 334) was the first fully human IgG2 monoclonal antibody developed for migraine prevention. Unlike antibodies that bind directly to CGRP, erenumab selectively blocks the canonical CGRP receptor, thereby preventing receptor activation and subsequent migraine-related signaling.

Several randomized controlled trials (RCTs) have evaluated the efficacy and safety of erenumab in patients with episodic and chronic migraine. However, differences in study populations, migraine subtype, dosing regimens, outcome definitions, and analytical approaches have contributed to variability in the available evidence. In addition, overlapping publications and multi-arm trials may complicate quantitative synthesis if participants are not appropriately accounted for. Although previous randomized trials and systematic reviews have established the efficacy and safety of erenumab, differences in study populations, outcome definitions, dosing regimens, and analytical approaches warrant an updated quantitative synthesis using independent placebo-controlled trials and outcome-specific analyses.

Therefore, the present systematic review and meta-analysis aimed to quantitatively evaluate the efficacy and safety of erenumab compared with placebo in patients with episodic or chronic migraine, focusing on the proportion of patients achieving a ≥50% reduction in monthly migraine days, change in monthly migraine days from baseline, and the incidence of overall and serious adverse events. Multi-arm trials were handled to avoid double-counting of participants, while overlapping or subgroup publications were not treated as independent trials.

This work was previously presented as a paper at the 27th International Student Congress of (bio)Medical Sciences (ISCOMS), held on June 4, 2020.

## Review

Methodology

Literature Search Strategy

This systematic review and meta-analysis was conducted in accordance with the PRISMA 2020 guidelines. The literature search was conducted from January 1, 2000, through January 31, 2026, using PubMed Central (PMC), the Cochrane Library, ClinicalTrials.gov, and Google Scholar. Searches were performed using combinations of terms related to migraine and erenumab/AMG 334. Google Scholar and ClinicalTrials.gov were additionally searched to identify potentially relevant studies and ongoing or unpublished evidence. The database-specific search strategies and numbers of records retrieved are presented in Table 1.

**Table 1:** Comprehensive literature search strategy used for the identification of randomized controlled trials evaluating the efficacy and safety of erenumab for migraine prevention The table summarizes the database-specific search strategies, search period, and number of records retrieved during the systematic literature search conducted from January 2000 through January 2026. Searches were performed in PMC, the Cochrane Library, ClinicalTrials.gov, and Google Scholar using combinations of terms related to migraine and erenumab/AMG 334. A total of 3,012 records were identified, including 2,992 records from the four electronic sources searched and 20 records identified through other sources. The detailed search strategies are presented to facilitate reproducibility of the literature search. PMC: PubMed Central; CGRP: Calcitonin gene-related peptide; RCT: Randomized controlled trial.

Database/Source	Search strategy	Search period	Records retrieved
PubMed Central (PMC)	((migraine*[tiab] OR "migraine disorder"[tiab] OR "migraine disorders"[tiab]) AND (erenumab[tiab] OR "AMG 334"[tiab])) AND 2000/01/01:2026/01/31[dp]	January 2000–January 2026	640
Cochrane Library	(migraine OR "migraine disorder*") AND (erenumab OR "AMG 334")	January 2000–January 2026	366
ClinicalTrials.gov	(migraine OR "migraine disorder") AND (erenumab OR "AMG 334")	January 2000–January 2026	66
Google Scholar	"migraine" AND ("erenumab" OR "AMG 334")	January 2000–January 2026	1,920
Total	—	—	2,992

Several publications related to the included erenumab trials reported outcomes that were not eligible for the primary quantitative synthesis. These included studies focusing primarily on patient-reported outcomes, migraine-related disability, and quality of life; subgroup analyses of previously included trials; and phase I studies primarily evaluating safety, tolerability, or pharmacokinetic characteristics [9-12]. Although these publications provide additional information regarding the clinical effects and longer-term use of erenumab, they were not considered independent RCTs for the present meta-analysis. Accordingly, overlapping or subgroup publications were excluded to avoid double-counting of participants, while studies reporting outcomes that could not be quantitatively harmonized with the predefined efficacy and safety outcomes were excluded from the quantitative synthesis. Following removal of duplicate records, two reviewers independently screened the titles and abstracts of the retrieved records. Full-text articles considered potentially eligible were subsequently assessed against the predefined inclusion and exclusion criteria. Any discrepancies between reviewers were resolved through discussion and consensus.

Study Selection and Eligibility Criteria

RCTs evaluating the efficacy and/or safety of erenumab compared with placebo for migraine prevention were eligible for inclusion. Eligible studies were required to use a randomized allocation procedure and a concurrent placebo control. Trials without a concurrent placebo-controlled randomized comparison were excluded from the primary quantitative synthesis. The predefined eligibility criteria for study selection are presented in Table 2.

**Table 2:** Inclusion and exclusion criteria applied for the selection of studies in the meta-analysis Studies were screened using predefined eligibility criteria established before the literature search. Only randomized controlled trials that compared erenumab with placebo in adults with episodic or chronic migraine and reported at least one predefined efficacy or safety outcome were eligible for inclusion. Studies were excluded if they had an inadequate follow-up duration, insufficient quantitative data for pooled analysis, a non-randomized study design, were conference abstracts without complete results, or were conducted in animal or other preclinical models.

Inclusion criteria	Exclusion criteria
Randomized, placebo-controlled trials evaluating the efficacy and/or safety of erenumab for migraine prevention	Follow-up duration < 7 days
Adult patients diagnosed with episodic or chronic migraine	Treatment duration < 3 months for the primary efficacy analysis
Comparison of erenumab with placebo	Studies evaluating behavioral or other non-pharmacological interventions without a relevant erenumab–placebo comparison
Studies reporting at least one predefined efficacy outcome, including monthly migraine days or ≥50% responder rate	Observational studies, case reports, case series, systematic reviews, narrative reviews, editorials, letters, and commentaries
Studies reporting at least one predefined safety outcome, including adverse events and/or serious adverse events	Conference abstracts without sufficient quantitative outcome data
Studies published from January 2000 through the date of the final literature search	Animal, laboratory, or preclinical studies
No restriction based on country or publication language	Studies with insufficient quantitative data to calculate an effect estimate
Studies providing sufficient quantitative data for meta-analysis	Duplicate publications or secondary/subgroup reports based on an already included trial population, unless they provided unique eligible outcome data
Studies reporting outcomes at a prespecified follow-up point suitable for quantitative synthesis	Studies not reporting any predefined efficacy or safety outcome of interest

Interventions

The intervention of interest was erenumab, administered as a monthly subcutaneous injection for migraine prevention, at any dose evaluated in eligible RCTs, including approved and investigational regimens. In all included studies, participants assigned to the control arm received placebo administered according to the respective trial protocol.

Outcome Measures

The analysis included studies involving adults diagnosed with either episodic migraine or chronic migraine. The primary efficacy outcome was the proportion of participants who achieved a ≥50% reduction in monthly migraine days during weeks 9-12 after treatment initiation, a widely accepted indicator of clinically meaningful improvement.

Secondary efficacy outcomes included the change in monthly migraine days from baseline. Safety outcomes comprised the incidence of overall adverse events and serious adverse events reported during the study period.

Data Extraction

Two reviewers independently extracted data using a predefined data collection form. Information obtained from each study included author details, study design, participant characteristics, intervention dose, comparator, duration of follow-up, and reported efficacy and safety outcomes. Efficacy variables included the ≥50% responder rate and change in monthly migraine days, whereas safety variables included the frequency of overall and serious adverse events. Studies that did not provide sufficient quantitative data for meta-analysis were excluded from pooled statistical analyses.

The total sample size refers to all participants randomized in the six independent trials. Outcome-specific denominators varied because individual trials used different analysis populations for efficacy and safety outcomes, reflecting outcome availability, post-randomization measurements, and the respective efficacy or safety analysis sets. Therefore, pooled denominators for individual outcomes do not necessarily equal the total number randomized across all included trials.

Assessment of Risk of Bias

The methodological rigor of each included RCT was assessed using the Cochrane Risk of Bias 2 (RoB 2) tool. The assessment covered five domains: bias arising from the randomization process; bias due to deviations from intended interventions; bias due to missing outcome data; bias in measurement of the outcome; and bias in selection of the reported result. Each domain was classified as low risk of bias, some concerns, or high risk of bias according to the RoB 2 framework. The assessments considered the adequacy of randomization and allocation procedures, the maintenance of blinding, adherence to assigned interventions, the completeness of outcome data, the appropriateness of outcome measurement, and potential selective reporting of results. Industry funding and conflicts of interest were considered as contextual factors but were not treated as independent RoB 2 domains. Overall risk-of-bias judgments were assigned based on the domain-level assessments.​​​​​​

Statistical Analysis

Continuous outcomes measured on the same scale were analyzed using mean differences (MDs) with 95% confidence intervals (CIs), whereas dichotomous outcomes were summarized as risk ratios (RRs) with corresponding 95% CIs. Given the expected variability in participant characteristics, treatment doses, and study methodologies, random-effects models were used to pool effect estimates throughout the analysis.

Summary Measures

The overall treatment effects are reported with 95% CIs. Forest plots were generated to illustrate pooled effect estimates across studies, while funnel plots were used to assess potential publication bias. All statistical analyses were conducted using Review Manager (RevMan®) version 5.3 (The Cochrane Collaboration, London, UK), and statistical significance was defined as a two-sided p-value < 0.05.

Results

Baseline Characteristics

A total of 3,012 records were identified, including 2,992 from electronic database and other electronic source searches and 20 from other sources. After removal of duplicate records, 1,310 records remained for title and abstract screening. Of these, 1,299 records were excluded, and 11 full-text articles were assessed for eligibility. Five articles were subsequently excluded: three reported only patient-reported outcomes, one was an ongoing Phase I study, and one was a subgroup or overlapping publication of an already included trial. Ultimately, six independent RCTs, involving 3,403 participants, were included in both the qualitative and quantitative syntheses (meta-analysis). The baseline characteristics of the included studies are summarized in Table 3, and the study selection process is illustrated in Figure 1.

**Table 3:** Baseline details of the studies included for meta-analysis The table summarizes the key characteristics of the six RCTs evaluating the efficacy and safety of erenumab for migraine prevention. Information presented includes study author and year, study design, intervention and control groups, sample size, and corresponding reference number. Erenumab was administered as a monthly subcutaneous injection at doses ranging from 7 to 140 mg, while placebo was administered via monthly subcutaneous injection in the control groups. RCT: Randomized controlled trial.

Study	Study design	Intervention	Control	Sample size, n
Sun et al. (2016) [13]	Phase 2, randomized, double-blind, placebo-controlled	Subcutaneous erenumab 7, 21, or 70 mg once monthly	Subcutaneous placebo once monthly	483
Goadsby et al. (2017) [14]	Phase 3, randomized, double-blind, placebo-controlled	Subcutaneous erenumab 70 or 140 mg once monthly	Subcutaneous placebo once monthly	955
Tepper et al. (2017) [15]	Phase 2, randomized, double-blind, placebo-controlled	Subcutaneous erenumab 70 or 140 mg once monthly	Subcutaneous placebo once monthly	667
Dodick et al. (2018) [16]	Phase 3, randomized, double-blind, placebo-controlled	Subcutaneous erenumab 70 mg once monthly	Subcutaneous placebo once monthly	577
Reuter et al. (2018) [17]	Phase 3b, randomized, double-blind, placebo-controlled	Subcutaneous erenumab 140 mg once monthly	Subcutaneous placebo once monthly	246
Sakai et al. (2019) [18]	Phase 2, randomized, double-blind, placebo-controlled	Subcutaneous erenumab 28, 70, or 140 mg once monthly	Subcutaneous placebo once monthly	475

**Figure 1:**
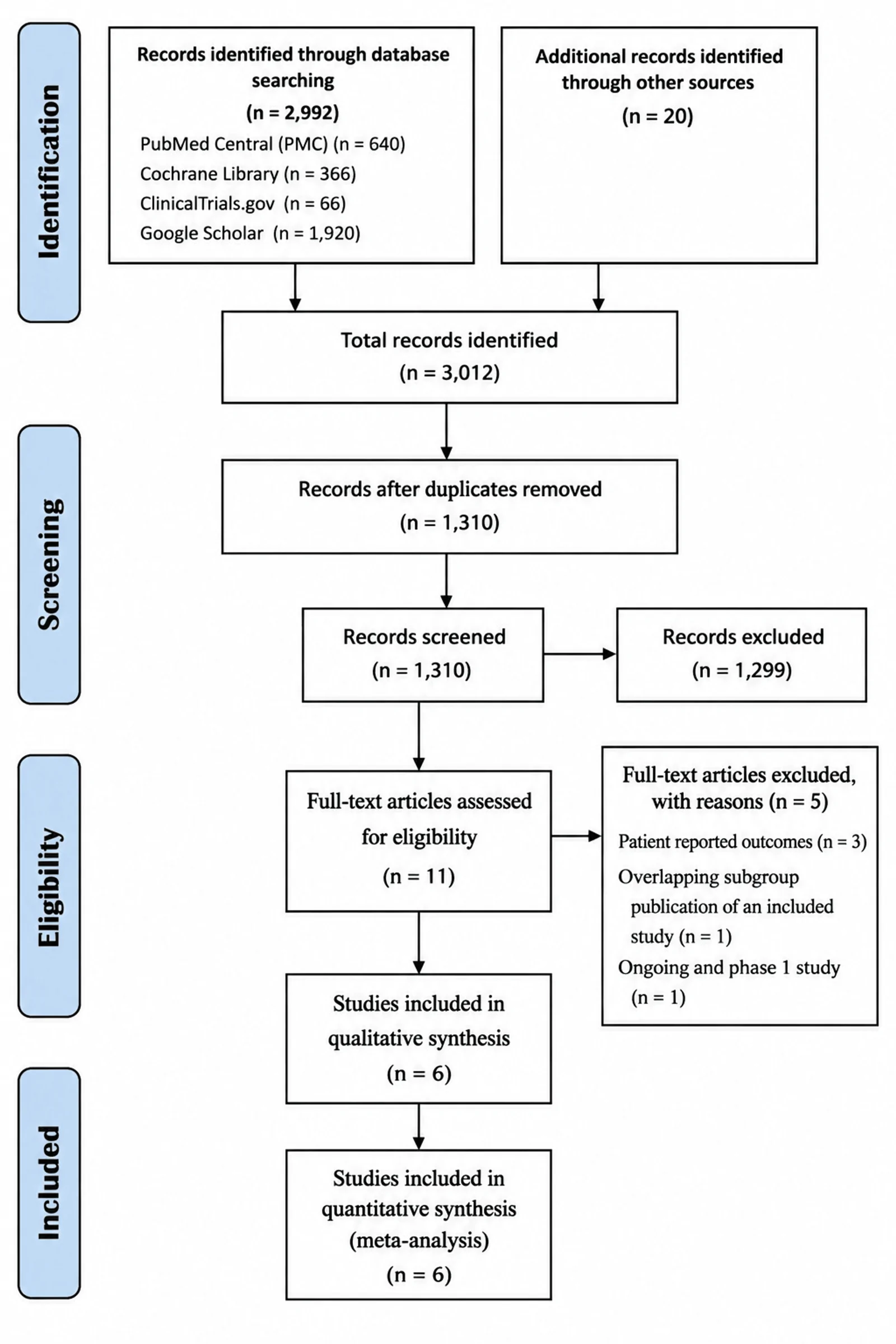
PRISMA flow diagram of included studies A total of 3,012 records were identified, including 2,992 records from the four electronic sources searched and 20 records identified through other sources. After removal of duplicate records, 1,310 unique records remained for title and abstract screening. Of these, 1,299 records were excluded because they did not meet the predefined eligibility criteria. The full texts of 11 potentially relevant articles were subsequently assessed for eligibility, of which five were excluded: three reported only patient-reported outcomes, one was an ongoing Phase I study, and one was a subgroup or overlapping publication of an already included trial. Consequently, six independent RCTs were included in both the qualitative synthesis and quantitative meta-analysis. RCT: Randomized controlled trials.

Risk of Bias Assessment

Risk of bias was assessed for all six included RCTs using the Cochrane RoB 2 tool. Across the five assessed domains, all six studies were judged to have low risk of bias for the randomization process, deviations from intended interventions, missing outcome data, and measurement of outcomes. Some concerns were identified for the selection of the reported result across the included studies. Consequently, the overall risk of bias was judged as “some concerns” for all six trials. The study-level assessments are presented in Table 4.

**Table 4:** Risk of bias assessment of the included randomized controlled trials using the Cochrane RoB 2 tool Risk of bias was assessed independently for each included randomized controlled trial (RCT) using the Cochrane Risk of Bias 2 (RoB 2) tool. The assessment evaluated five predefined domains: (1) bias arising from the randomization process, including the adequacy of sequence generation and allocation concealment; (2) bias due to deviations from intended interventions, including adherence to the assigned intervention and maintenance of blinding; (3) bias due to missing outcome data, including the extent and potential influence of participant attrition; (4) bias in measurement of the outcome, considering the appropriateness and consistency of outcome assessment and the potential influence of knowledge of intervention assignment; and (5) bias in selection of the reported result, including the possibility of selective reporting among multiple eligible analyses or outcome measurements. Each domain was judged as low risk of bias, some concerns, or high risk of bias, and an overall risk-of-bias judgment was assigned to each study according to the RoB 2 framework.

Study	Randomization process	Deviations from intended interventions	Missing outcome data	Measurement of outcome	Selection of reported result	Overall RoB
Sun et al. (2016) [13]	Low risk	Low risk	Low risk	Low risk	Some concerns	Some concerns
Goadsby et al. (2017) [14]	Low risk	Low risk	Low risk	Low risk	Some concerns	Some concerns
Tepper et al. (2017) [15]	Low risk	Low risk	Low risk	Low risk	Some concerns	Some concerns
Dodick et al. (2018) [16]	Low risk	Low risk	Low risk	Low risk	Some concerns	Some concerns
Reuter et al. (2018) [17]	Low risk	Low risk	Low risk	Low risk	Some concerns	Some concerns
Sakai et al. (2019) [18]	Low risk	Low risk	Low risk	Low risk	Some concerns	Some concerns

Primary Efficacy Endpoints

A ≥50% reduction in monthly migraine days at 12 weeks was reported across the included RCTs. Across the six independent trials, 792 of 2,039 participants (38.9%) receiving erenumab achieved the predefined responder outcome, compared with 307 of 1,289 participants (23.8%) receiving placebo. The random-effects model demonstrated that erenumab significantly increased the likelihood of achieving a ≥50% reduction in monthly migraine days compared with placebo (RR = 1.660, 95% CI: 1.364-2.020; p < 0.001). The corresponding fixed-effect model yielded an RR of 1.605 (95% CI: 1.435-1.797; p < 0.001). Between-study heterogeneity was moderate (Cochran’s Q = 12.95, df = 5; I² = 61.4%). The pooled estimates are presented in Table 5 and Figure 2.

**Table 5:** Relative risk of achieving a ≥50% reduction in monthly migraine days at 12 weeks with erenumab versus placebo Relative risk of the specified outcome among patients receiving erenumab compared with placebo across six independent randomized controlled trials. Events are presented as the number of participants experiencing the outcome over the total number of participants included in the respective efficacy or safety analysis. Study-specific relative risks (RRs) are presented with corresponding 95% confidence intervals (CIs). The pooled effect estimates and study weights are presented for fixed-effect and random-effects models. The pooled random-effects analysis demonstrated that erenumab significantly increased the likelihood of achieving a ≥50% reduction in monthly migraine days compared with placebo (RR = 1.66, 95% CI = 1.36–2.02; p < 0.001). Moderate between-study heterogeneity was observed (Cochran's Q = 12.95, df = 5; I² = 61.4%).

Study	Erenumab events/Total	Placebo events/Total	Relative risk (RR)	95% CI	Fixed-effect weight (%)	Random-effects weight (%)
Sun et al. (2016) [13]	108/296	43/144	1.222	0.912–1.636	14.84	17.89
Goadsby et al. (2017) [14]	294/630	84/316	1.756	1.435–2.147	31.21	22.57
Tepper et al. (2017) [15]	152/375	66/281	1.726	1.352–2.203	21.25	20.31
Dodick et al. (2018) [16]	112/282	85/288	1.346	1.070–1.692	24.09	21.09
Reuter et al. (2018) [17]	36/119	17/124	2.207	1.313–3.708	4.70	9.64
Sakai et al. (2019) [18]	90/337	12/136	3.027	1.714–5.344	3.92	8.50
Total (fixed-effect model)	792/2039	307/1289	1.605	1.435–1.797	100.00	—
Total (random-effects model)	792/2039	307/1289	1.660	1.364–2.020	—	100.00

**Figure 2:**
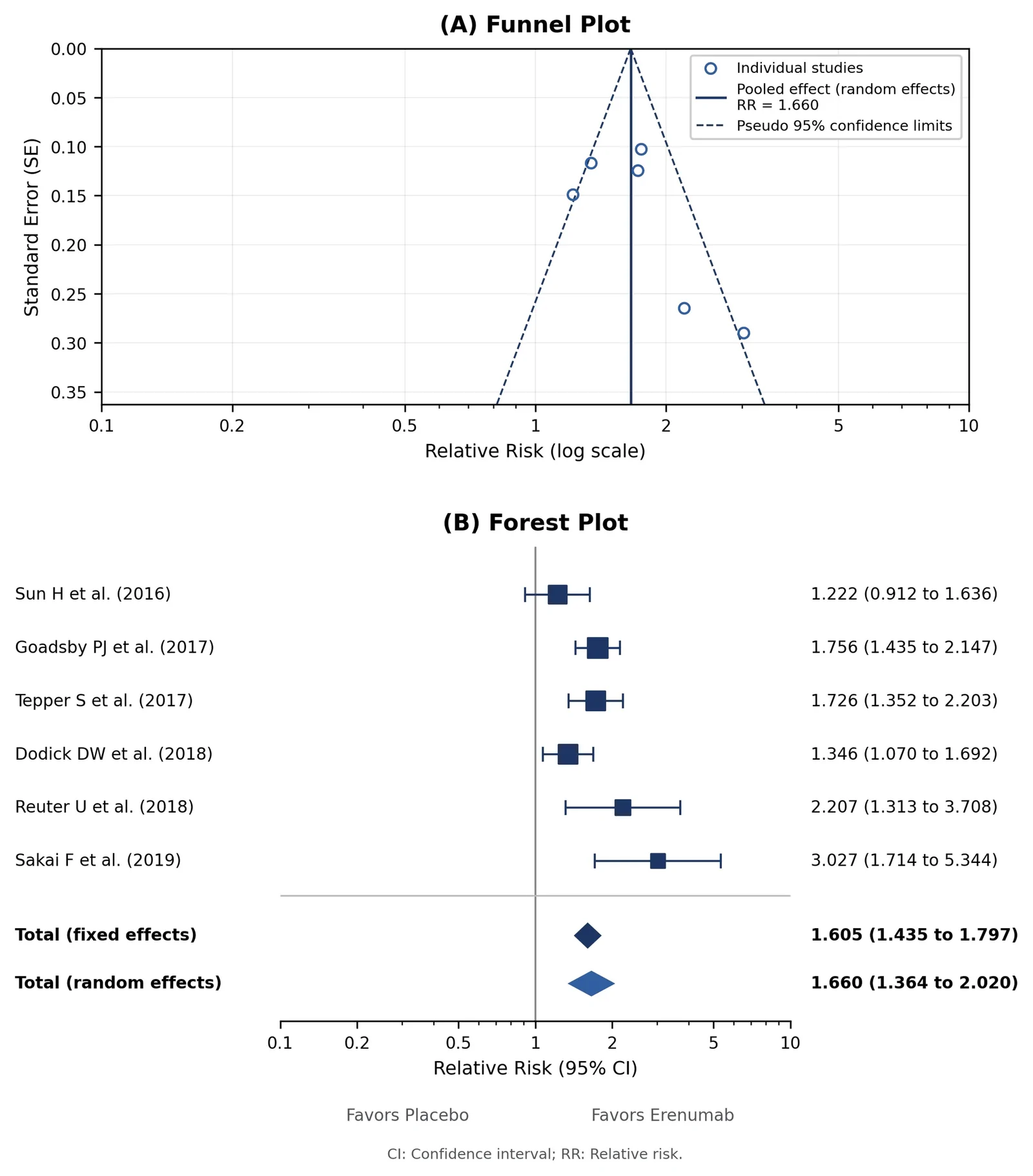
Funnel and forest plots assessing the efficacy of erenumab versus placebo in achieving a ≥50% reduction in monthly migraine days at 12 weeks (A) Funnel plot showing the distribution of six independent randomized controlled trials according to the relative risk (RR) of the specified outcome and its standard error (SE). The solid vertical line represents the pooled random-effects estimate (RR = 1.660), while the dashed lines indicate the pseudo-95% confidence limits. The plot did not show obvious asymmetry; however, interpretation of funnel-plot asymmetry was limited by the small number of included studies, and the findings should therefore be interpreted cautiously. (B) Forest plot showing the study-specific and pooled relative risks for the specified outcome in patients receiving erenumab compared with placebo. Squares represent individual study estimates, with horizontal lines indicating 95% confidence intervals (CIs); the diamonds represent the pooled fixed-effect and random-effects estimates. The random-effects model yielded a pooled RR of 1.660 (95% CI, 1.364–2.020).

Secondary events

The quantitative synthesis of six independent RCTs demonstrated a consistent reduction in monthly migraine days with erenumab compared with placebo. As shown in Table 6 and Figure 3, the individual study effects ranged from −0.36 to −2.46 days/month, with all studies favoring erenumab. The pooled random-effects analysis demonstrated a significantly greater reduction in monthly migraine days with erenumab than with placebo (MD: −1.51 days/month; 95% CI: −2.03 to −0.99; p < 0.001). There was substantial between-study heterogeneity (Cochran's Q = 21.19, df = 5, I² = 76.4%, τ² = 0.31). 

**Table 6:** Mean difference in the change in monthly migraine days from baseline between erenumab and placebo The table presents the quantitative synthesis of six independent randomized controlled trials evaluating erenumab for migraine prevention. Treatment effects are expressed as mean differences (MDs) in monthly migraine days, with corresponding standard errors (SEs) and 95% confidence intervals (CIs). For trials evaluating more than one erenumab dose, the eligible erenumab treatment arms were combined to provide a single comparison with the common placebo group, thereby avoiding double-counting of participants. A random-effects model was used for the pooled analysis. A negative MD indicates a greater reduction in monthly migraine days with erenumab than with placebo. The pooled random-effects estimate was −1.51 days/month (95% CI: −2.03 to −0.99). The analysis included six independent trials; the subgroup publication by Dodick et al. (2018) was not treated as an independent study to avoid potential duplication of participants from the parent trial [16]. For multi-arm trials, erenumab dose groups were combined before comparison with the shared placebo group.

Study	Erenumab dose(s) combined	Erenumab, n	Placebo, n	Mean difference (days/month)	Standard error	95% CI	Weight (%)
Sun et al. (2016) [13]	7, 21, and 70 mg	323	160	−0.36	0.379	−1.11 to 0.38	15.68
Goadsby et al. (2017) [14]	70 and 140 mg	630	316	−1.65	0.245	−2.13 to −1.17	19.26
Tepper et al. (2017) [15]	70 and 140 mg	360	267	−2.46	0.384	−3.21 to −1.70	15.55
Dodick et al. (2018) [16]	70 mg	268	270	−1.04	0.291	−1.61 to −0.47	18.05
Reuter et al. (2018) [17]	140 mg	118	120	−1.61	0.556	−2.70 to −0.52	11.47
Sakai et al. (2019) [18]	28, 70, and 140 mg	337	136	−1.89	0.216	−2.31 to −1.47	20.00
Overall random-effects model	—	—	—	−1.51	0.266	−2.03 to −0.99	100.00

**Figure 3:**
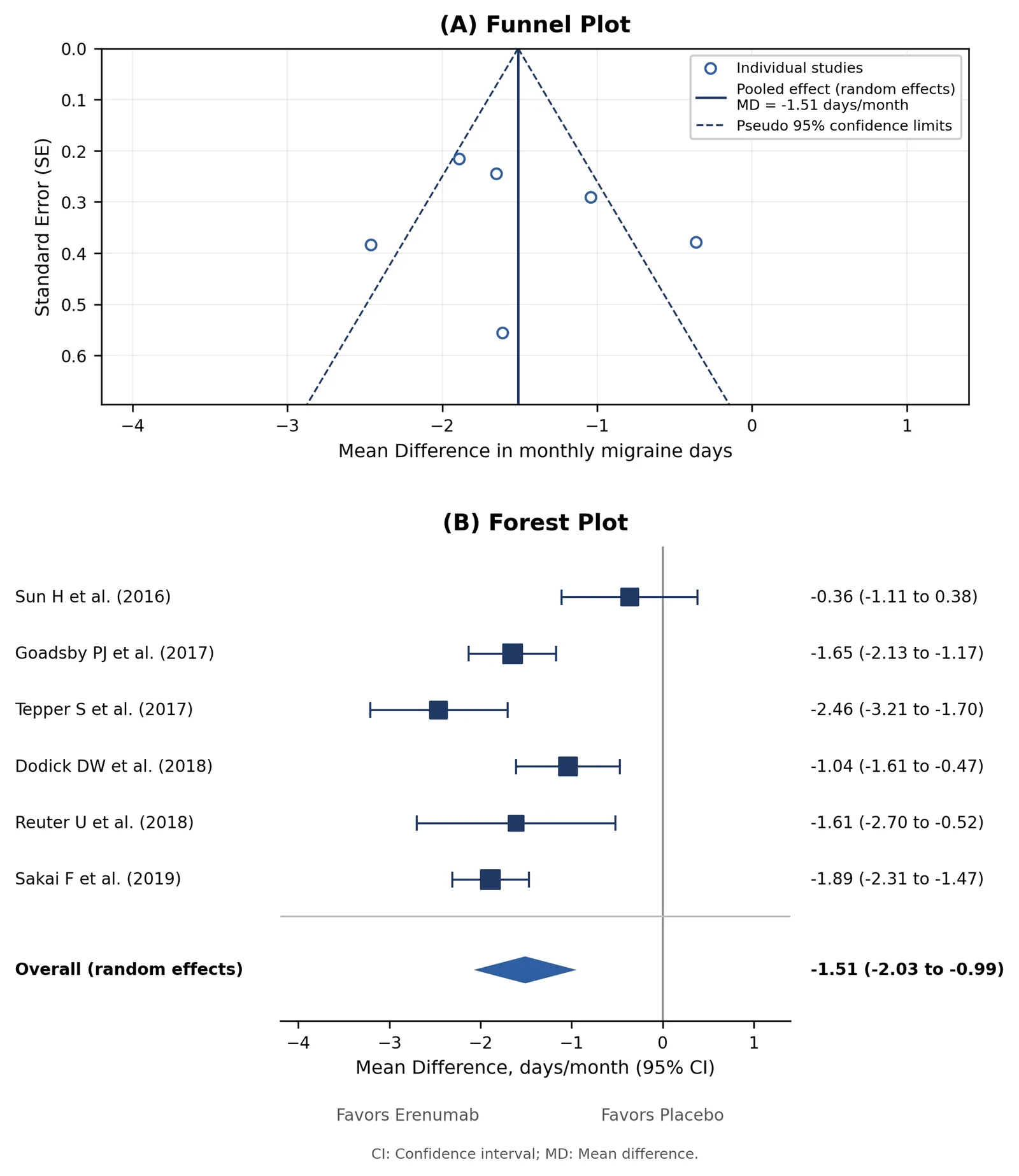
Meta-analysis of the effect of erenumab on monthly migraine days compared with placebo (A) Funnel plot showing the distribution of six independent randomized controlled trials according to the mean difference (MD) in change in monthly migraine days (MMD) from baseline and its standard error (SE). The solid vertical line represents the pooled random-effects estimate (MD = −1.51 days/month), while the dashed lines represent the pseudo-95% confidence limits. The plot did not show obvious asymmetry; however, interpretation of funnel-plot asymmetry was limited by the small number of included studies, and the findings should therefore be interpreted cautiously. (B) Forest plot showing study-specific and pooled effects of erenumab compared with placebo on change in MMD. Squares represent individual study estimates, with their size corresponding to the study weight; horizontal lines represent 95% confidence intervals. The diamonds represent the pooled estimates under fixed-effect and random-effects models. Negative MDs indicate a greater reduction in monthly migraine days with erenumab than with placebo. The random-effects model demonstrated a pooled MD of −1.51 days/month (95% CI, −2.03 to −0.99).

Safety Outcomes

(A) Adverse events: The analysis of overall adverse events included six independent RCTs. As shown in Table 7 and Figure 4, adverse events occurred in 1,094/2,070 (52.9%) of participants receiving erenumab and 710/1,303 (54.5%) of participants receiving placebo. The pooled random-effects analysis showed no statistically significant difference in the risk of adverse events (RR, 0.968; 95% CI, 0.894-1.048). The corresponding fixed-effect estimate was RR 0.959 (95% CI: 0.901-1.021). There was low-to-moderate heterogeneity among the studies (Cochran's Q = 7.49, df = 5, I² = 33.2%, τ² = 0.0032).

**Table 7:** Relative risk of erenumab vs placebo group in six RCTs for adverse effects at the end of 12 weeks Relative risk of adverse events among patients receiving erenumab compared with placebo across six independent randomized controlled trials. Events are presented as the number of participants experiencing at least one adverse event, divided by the total number of participants included in the respective safety analysis. Relative risks (RRs) are presented with corresponding 95% confidence intervals (CIs). Study-specific weights are shown for fixed-effect and random-effects models. The pooled effect was estimated using both fixed-effect and random-effects models. A relative risk <1.0 indicates a lower risk of adverse events with erenumab than with placebo, whereas a relative risk >1.0 indicates a higher risk with erenumab. The pooled random-effects analysis showed no statistically significant difference in the risk of adverse events between erenumab and placebo (RR, 0.968; 95% CI, 0.894–1.048).

Study	Erenumab events/Total	Placebo events/Total	Relative risk (RR)	95% CI	Fixed-effect weight (%)	Random-effects weight (%)
Sun et al. (2016) [13]	165/319	82/153	0.965	0.805–1.157	11.85	13.88
Goadsby et al. (2017) [14]	357/633	201/319	0.895	0.803–0.998	33.23	26.12
Tepper et al. (2017) [15]	171/378	110/282	1.160	0.965–1.393	11.63	13.69
Dodick et al. (2018) [16]	136/283	158/289	0.879	0.749–1.032	15.21	16.54
Reuter et al. (2018) [17]	65/119	67/124	1.011	0.803–1.273	7.35	9.60
Sakai et al. (2019) [18]	230/338	92/136	1.006	0.877–1.154	20.74	20.18
Overall fixed-effect model	1094/2070	710/1303	0.959	0.901–1.021	100.00	—
Overall random-effects model	1094/2070	710/1303	0.968	0.894–1.048	—	100.00

**Figure 4:**
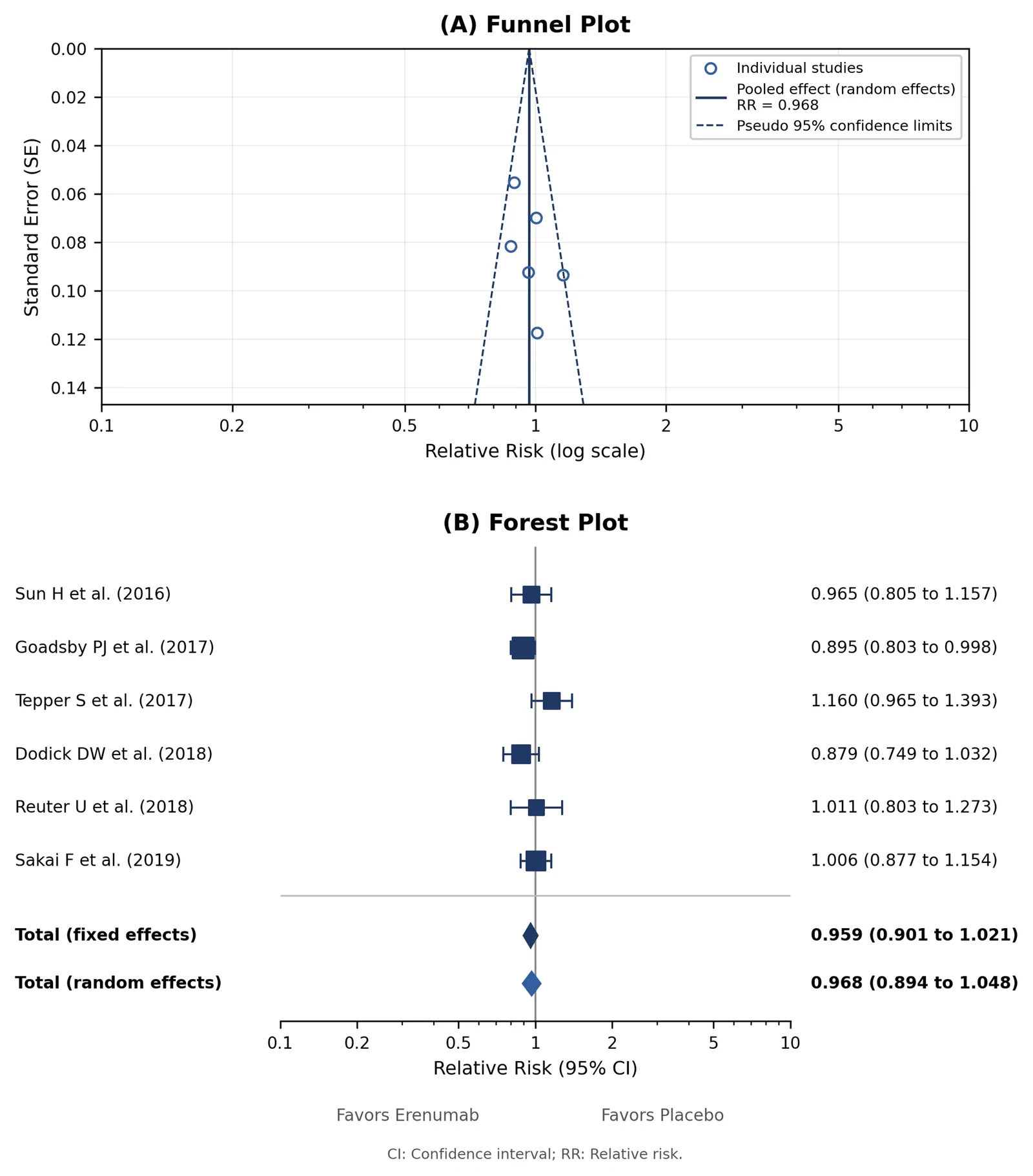
Funnel and forest plot results of adverse effects of erenumab vs placebo studies at 12 weeks (A) Funnel plot assessing potential publication bias among the randomized controlled trials included in the meta-analysis of adverse events. The distribution of studies around the pooled effect estimate did not show obvious asymmetry; however, interpretation of funnel-plot asymmetry was limited by the small number of included studies, and the findings should therefore be interpreted cautiously. (B) Forest plot presenting individual study and pooled RRs with 95% CIs for the occurrence of adverse events in patients receiving erenumab compared with placebo. Squares represent study-specific effect estimates, with the size of each square proportional to the study weight, while horizontal lines indicate 95% confidence intervals. Diamonds represent the pooled estimates obtained using fixed-effect and random-effects models. The pooled analysis demonstrated no statistically significant difference in the incidence of adverse events between the erenumab and placebo groups, indicating a comparable safety profile.

(B) Serious adverse events: As presented in Table 8 and Figure 5, serious adverse events occurred in 32 of 2,070 participants (1.5%) receiving erenumab and 25 of 1,303 participants (1.9%) receiving placebo. The pooled fixed-effect and random-effects models yielded identical estimates (RR = 0.791, 95% CI: 0.463-1.349). The pooled analysis showed no statistically significant difference in the risk of serious adverse events between erenumab and placebo, as the 95% CI included 1.0 (p = 0.389). No statistical heterogeneity was detected among the six studies (Cochran’s Q = 3.46, df = 5; I² = 0%; τ² = 0.00).

**Table 8:** Relative risk of erenumab vs placebo group in six randomized controlled trials for serious adverse effects at the end of 12 weeks Relative risk of serious adverse events among participants receiving erenumab compared with placebo across six independent randomized controlled trials. Events are presented as the number of participants experiencing a serious adverse event over the total number included in the respective analysis. Study-specific relative risks (RRs) are presented with corresponding 95% confidence intervals (CIs), together with study weights under fixed-effect and random-effects models. The pooled analysis showed no statistically significant difference in the risk of serious adverse events between erenumab and placebo (RR = 0.79, 95% CI = 0.46–1.35; p = 0.389). An RR > 1 indicates a higher risk of serious adverse events with erenumab, whereas an RR < 1 indicates a lower risk compared with placebo. The analysis included six independent randomized controlled trials; Dodick et al. (2018) was excluded because it represented a subgroup analysis of an already included trial and was therefore not treated as an independent study. No statistical heterogeneity was detected among the included studies (Cochran’s Q = 3.46, df = 5; I² = 0%; τ² = 0.00).

Study	Erenumab events/Total	Placebo events/Total	Relative risk (RR)	95% CI	Fixed-effect weight (%)	Random-effects weight (%)
Sun et al. (2016) [13]	2/319	0/153	2.406	0.116–49.819	3.11	3.11
Goadsby et al. (2017) [14]	14/633	7/319	1.008	0.411–2.472	35.45	35.45
Tepper et al. (2017) [15]	8/378	7/282	0.853	0.313–2.324	28.39	28.39
Dodick et al. (2018) [16]	3/283	6/289	0.511	0.129–2.022	15.07	15.07
Reuter et al. (2018) [17]	2/119	1/124	2.084	0.191–22.682	5.01	5.01
Sakai et al. (2019) [18]	3/338	4/136	0.302	0.068–1.331	12.97	12.97
Total (fixed-effect model)	32/2070	25/1303	0.791	0.463–1.349	100.00	—
Total (random-effects model)	32/2070	25/1303	0.791	0.463–1.349	—	100.00

**Figure 5:**
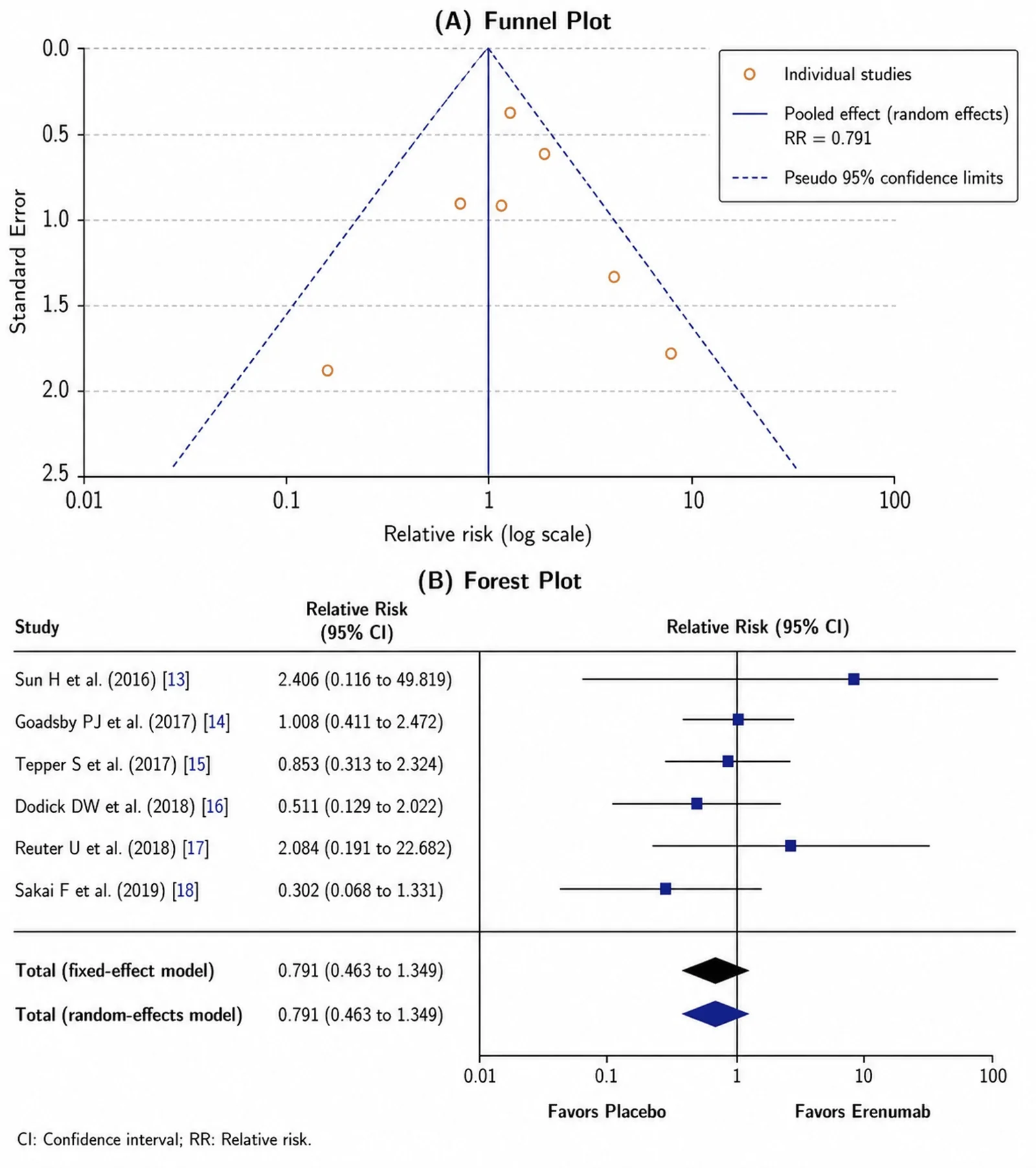
Funnel and forest plot results of serious adverse effects of erenumab vs placebo studies at 12 weeks (A) Funnel plot showing the distribution of six independent randomized controlled trials according to the relative risk (RR) of the specified outcome and its standard error (SE). The solid vertical line represents the pooled random-effects estimate (RR = 0.791), while the dashed lines represent the pseudo-95% confidence limits. The plot did not show obvious asymmetry; however, interpretation of funnel-plot asymmetry was limited by the small number of included studies, and the findings should therefore be interpreted cautiously. (B) Forest plot showing the study-specific and pooled relative risks for the specified outcome in participants receiving erenumab compared with placebo. Squares represent individual study estimates, with horizontal lines indicating 95% confidence intervals (CIs); the diamonds represent the pooled fixed-effect and random-effects estimates. The pooled fixed-effect and random-effects estimates were both RR 0.791 (95% CI, 0.463–1.349).

Discussion

This meta-analysis shows that erenumab provides clinically meaningful benefits for migraine prevention. Across six independent RCTs, erenumab significantly increased the proportion of patients achieving a ≥50% reduction in monthly migraine days compared with placebo (RR, 1.66; 95% CI, 1.36-2.02). Erenumab also produced a significantly greater reduction in monthly migraine days, with a pooled mean difference of −1.51 days/month (95% CI: −2.03 to −0.99). These efficacy benefits were not accompanied by a significant increase in overall adverse events or serious adverse events. Overall adverse events were comparable between erenumab and placebo (RR, 0.97; 95% CI, 0.89-1.05), while serious adverse events were also not significantly different (RR, 0.79; 95% CI, 0.46-1.35). Thus, the findings support a favorable efficacy and safety profile for erenumab in migraine prevention.

The efficacy observed in this analysis is consistent with the work of Markham, who summarized evidence from phase II and phase III clinical trials demonstrating that erenumab significantly reduced migraine frequency and subsequently received regulatory approval as the first fully human monoclonal antibody directed against the CGRP receptor for migraine prevention [19]. The increased responder rate and reduction in monthly migraine days observed in the present pooled analysis further support the clinical benefit demonstrated in these pivotal trials.

Our results are also comparable with those reported by Schwedt et al., who showed that patients receiving erenumab experienced measurable clinical improvement within the first week of therapy in both episodic and chronic migraine [20]. Although the present analysis evaluated outcomes at approximately 12 weeks, the significant reduction in monthly migraine days across the six included trials is consistent with the early clinical improvement previously described.

Similarly, Lattanzi et al. reported that monthly administration of erenumab at doses of both 70 and 140 mg significantly reduced monthly migraine days and migraine-specific medication use without increasing treatment-related adverse events [21]. The present analysis yielded comparable findings, demonstrating a significant overall reduction in monthly migraine days and no significant increase in overall or serious adverse events compared with placebo. These findings support the favorable safety profile of erenumab observed in previous clinical investigations.

Comparable evidence has also been reported by Gui et al., whose meta-analysis of eight RCTs demonstrated significant reductions in migraine frequency across multiple dosing regimens, with the largest treatment effect observed at the 140 mg dose [22]. In the present analysis, the pooled treatment effect was also favorable for erenumab; however, the current revised quantitative synthesis does not provide a valid pooled comparison between the 70-mg and 140-mg doses. Therefore, a conclusion regarding superiority of either approved dose should not be drawn from the present overall analysis. Differences in sample size, study populations, baseline disease characteristics, and treatment exposure may contribute to variation in treatment effects between individual trials.

The findings of Zhu et al. further support the results of the present study. Their pooled analysis demonstrated higher rates of achieving a ≥50% reduction in monthly migraine days among patients treated with erenumab, along with reductions in monthly migraine days and migraine-specific medication use, without an increase in overall or serious adverse events [23]. These observations closely correspond to the present findings, which demonstrate a significantly higher responder rate and a reduction in monthly migraine days, without a statistically significant increase in overall or serious adverse events.

The comparative effectiveness of different erenumab doses has also been investigated by Yang et al., who suggested that the 140 mg regimen may provide greater reductions in migraine burden than the 70 mg dose [24]. Although the present analysis included trials evaluating both doses, the revised quantitative synthesis combined eligible erenumab treatment arms within multi-arm trials to provide a single comparison with placebo. Consequently, the present pooled analysis should be interpreted as an estimate of the overall efficacy of erenumab rather than evidence that 140 mg is superior to 70 mg. The apparent differences between individual trials may reflect variations in patient characteristics, migraine severity, study design, and prior exposure to preventive treatment.

Additional support comes from the recent GRADE (Grading of Recommendations, Assessment, Development, and Evaluations)-based systematic review and meta-analysis by Haseeb et al., which included 20 RCTs involving 5,212 participants [25]. Their study demonstrated significant improvements in migraine frequency, migraine-related disability, medication use, and responder rates following erenumab therapy. Consistent with their findings, the present analysis demonstrated a significant improvement in the ≥50% responder outcome and a significant reduction in monthly migraine days, while overall and serious adverse events did not differ significantly between erenumab and placebo. Collectively, these findings strengthen the evidence supporting erenumab as an effective preventive treatment for migraine.

Taken together, the findings of this meta-analysis are broadly consistent with previous randomized trials and systematic reviews. The significant improvement in the clinically meaningful ≥50% responder outcome and the additional reduction of approximately 1.5 migraine days per month compared with placebo support the efficacy of erenumab for migraine prevention. Importantly, the absence of a statistically significant increase in overall adverse events or serious adverse events supports its favorable short-term safety profile. However, the substantial heterogeneity observed for the change in monthly migraine days (I² = 76.4%) indicates that the magnitude of benefit varied considerably across studies. In contrast, heterogeneity was lower for overall adverse events (I² = 33.2%) and absent for serious adverse events (I² = 0%). The funnel plot did not show obvious asymmetry; however, the assessment of publication bias was limited by the small number of included studies, and the findings should therefore be interpreted with caution. These differences suggest that clinical and methodological variation between trials may influence the magnitude of efficacy benefit more than the observed safety outcomes.

Limitations

Several limitations should be considered when interpreting the findings of this meta-analysis. First, only six independent RCTs were included in the quantitative synthesis, limiting statistical power for subgroup analyses and reducing the ability to detect uncommon adverse events. The study reported by Dodick et al. was not considered an independent study because of its overlap with the parent trial, thereby avoiding potential double-counting of participants [16]. Second, most included studies assessed outcomes over approximately 12 weeks, limiting conclusions regarding the long-term efficacy and safety of erenumab. Although longer-term extension studies have reported sustained efficacy, these were not incorporated into the present short-term randomized quantitative synthesis. Third, substantial heterogeneity was observed for the change in monthly migraine days (I² = 76.4%), suggesting important differences in treatment effects across studies. Differences in migraine subtype, baseline disease severity, sample characteristics, dosing regimens, and previous preventive treatment exposure may have contributed to this heterogeneity. In contrast, heterogeneity was lower for overall adverse events (I² = 33.2%) and absent for serious adverse events (I² = 0%). Fourth, patient-reported outcomes, quality-of-life measures, and functional disability scores were not quantitatively synthesized because of inconsistent reporting across the included trials. The relatively small number of independent studies also limits the reliability of formal assessments of publication bias. Finally, this review relied on published aggregate data rather than individual patient-level datasets. Consequently, more detailed analyses according to age, migraine subtype, baseline migraine frequency, previous preventive treatment failure, and other potential treatment-effect modifiers could not be performed. Additionally, because the revised primary quantitative synthesis appropriately combined erenumab treatment arms within multi-arm trials, the present pooled estimates do not establish comparative superiority between the 70-mg and 140-mg doses.

## Conclusions

The findings of this meta-analysis indicate that erenumab is an effective preventive treatment for patients with EM or CM. Compared with placebo, erenumab significantly increased the proportion of patients achieving a ≥50% reduction in monthly migraine days and produced a significantly greater reduction in monthly migraine days. These efficacy benefits were not accompanied by a statistically significant increase in overall or serious adverse events, supporting a favorable short-term safety profile in the included trials. However, substantial heterogeneity was observed in the reduction of monthly migraine days, indicating variability in treatment effects across studies. Overall, the available evidence supports erenumab as an effective mechanism-based preventive therapy for migraine. Further well-designed studies with longer follow-up are warranted to establish its long-term efficacy and safety and to better characterize its role in migraine prevention.
